# Incidence of Radial Artery Occlusion in Patients Undergoing Percutaneous Coronary Intervention via Trans Radial Access

**DOI:** 10.12669/pjms.39.2.7219

**Published:** 2023

**Authors:** Syed Naseem Bukhari, Imran Javed, Muhammad Usman

**Affiliations:** 1 Syed Naseem Bukhari, MBBS, FCPS, Assistant Professor, Department of Cardiology, Ch, Pervaiz Elahi Institute of Cardiology, Multan, Pakistan; 2 Imran Javed, MBBS, FCPS, Assistant Professor, Nishtar Medical University & Hospital Multan, Pakistan; 3 Muhammad Usman, MBBS, Department of Cardiology, Nishtar Medical University & Hospital Multan, Pakistan

**Keywords:** Radial Artery, Occlusion, Percutaneous coronary artery occlusion

## Abstract

**Objective:**

To determine the incidence of radial artery occlusion (RAO) in patients undergoing percutaneous coronary intervention (PCI) via trans-radial access (TRA).

**Method:**

A descriptive study was carried out at the Department of interventional cardiology, Chaudhary Pervaiz Elahi Institute of Cardiology, Multan from 30-April 2019 to 30-October 2020. One hundred and twenty-five patients, who underwent PCI by TRA were selected for this study. The presence of Radial artery occlusion was noted 24 hours after the procedure by Doppler ultrasonography. SPSS version 23 was used for data analysis. A Chi-square test was applied. P-value < 0.05 was taken as statistically significant.

**Results:**

Gender distribution revealed 109 (87.2%) males and 16 (12.8%) females. The mean age of the patients was 65.22 ± 11.54 years. The mean BMI of the patients was 29.93±4.87 kg/m^2^. 84 (67.2%) patients were hypertensive, 40 (32%) patients were diabetics, 22 (17.6%) patients were smoker and 24 (19.2%) patients were having dyslipidemia. RAO after 24 hours was found in 5(4.0%) patients.

**Conclusion:**

Radial artery occlusion is a common complication of trans-radial access so radial artery patency must be checked before using it for transcatheter procedures.

## INTRODUCTION

In recent times, the radial artery is becoming popular for its use in transcatheter coronary procedures as a default access site not only in the developed western world but also in the developing countries of Asia.^1^-^3^ Trans Radial Access (TRA) has shown fewer complications like retroperitoneal bleeding and access site vascular complications like hematoma as compared to trans femoral access and is more comfortable for the patient as it allows early mobilization.^4^,^5^ But it doesn’t mean that TRA is without any complications. TRA is not only technically difficult but it takes a longer time to learn and leads to radial artery complications in especially in women and older patients.^6^,^7^

Radial artery occlusion is a serious post-procedure complication of trans-radial access that may cause critical ischemia of the used limb. Once the radial artery is occluded permanently, it cannot be used in future for percutaneous coronary intervention (PCI), fistula formation in hemodialysis patients or as a conduit for coronary bypass grafting.^8^ The incidence of RAO in literature is reported to be as low as 0.8% to as high as 38%.^9^ Aykan et al. found a 5.5% frequency of RAO after PCI in their study patients.^10^ A systematic review has reported a 0.8% to 30% frequency of RAO in coronary artery disease patients after PCI.^11^

There is high variability in the reported literature regarding the frequency of RAO after PCI. In addition, research has revealed demographic features such as gender, age, diabetes and body mass index (BMI) may affect the frequency of RAO.^12^ The aim of the proposed study was to find out the incidence of RAO after PCI. The results of this study will help us to determine the true prevalence of RAO in our population.

## METHOD

It is a descriptive Case Series study carried out from 30-April 2019 to 30-October 2020 at the department of interventional cardiology Chaudhary Pervaiz Elahi Institute of Cardiology Multan. A total of 125 patients were selected for the study. The study was approved by the ethics committee ref# 12-48 dated 13-03-2019 and consent was taken from all the included patients. The sample size for this study was calculated by taking the expected frequency of RAO in 5.5% of patients after PCI, at the desired precision level of 4.0% and 95% confidence.

A consecutive non-probability sampling technique was used. Patients of both genders in the age group 30 to 70 years who had undergone PCI via TRA were part of this study. Patients who have a history of PCI in past using TRA were not included as subjects. Percutaneous coronary intervention via TRA in all patients was performed by a senior cardiologist with three years of post-fellowship experience. Diagnosis of RAO was made by using the ultrasonic Doppler signal flow detector seven MHz (FD1, Huntleigh, Sonicaid) probe. A probe was placed above to access the site of the radial artery in the resting and extended position of the forearm. RAO was defined as the absence of any Doppler signal flow.

Doppler ultrasonography was performed 24 hours after the procedure for accessment of RAO. Confounder variables e.g. patient’s age, gender, diabetes, hypertension, smoking, dyslipidemia and BMI were also noted. The collected information was analyzed with SPSS version 16. Mean and standard deviation was calculated for continuous variables like age, height, weight and BMI. Frequency and percentage were calculated for categorical variables i.e. gender, diabetes, hypertension, smoking, dyslipidemia and RAO at 24 hours. Stratification of confounder variables e.g. patient’s age, gender, BMI, hypertension, smoking, dyslipidemia and diabetes was done. A Chi-square test was applied for stratification. P-value of < 0.05 was taken as a significant difference.

## RESULTS

A total of 125 patients were a part of the study including 109 (87.2%) men and 16 (12.8%) women. The mean age of the patients was 65.22±11.54 years, mean weight was 68.81±15.55 kg, mean height was 163.54 ± 9.07 cm and mean BMI was 29.93 ± 4.87 kg/m^2^. 84 (67.2%) patients were hypertensive, 40 (32%) patients were diabetics, 22 (17.6%) patients were smoker and 24 (19.2%) patients were having dyslipidemia.

RAO after 24 hours was found in five (4%) patients (Table-I). To check the effect modification, the chi-square test was applied. The relation of RAO with age, gender and BMI is shown in Tables-II, III and IV.

**Table-I:** RAO after 24 hours among the patients.

RAO after 24 hours	n (%)
Yes	n=5 (4%)
No	n=120 (96%)
Total	n=125 (100%)

**Table-II:** Association of RAO after 24 hours with age distribution.

Age distribution	RAO after 24 hours		P-value

	Yes	No	
30-50 years	2	53	0.854
51-70 years	3	67	
Total	5	120	

**Table-III:** Association of RAO after 24 hours with a gender distribution.

Gender distribution	RAO after 24 hours		P-value

	Yes	No	
Male	3	106	0.063
Female	2	14	
Total	5	120	

**Table-IV:** Association of RAO after 24 hours with BMI distribution.

BMI distribution	RAO after 24 hours		P-value

	Yes	No	
27-29 kg/m^2^	2	63	0.773
<29 kg/m^2^	3	57	
Total	5	120	

## DISCUSSION

This study was conducted to access the incidence of RAO after PCI in Pakistan. An occluded artery after PCI can result in misdiagnosis of RAO like in Brancheau et al, RAO was accessed by the absence of a radial pulse. 3.7% of patients did not have a radial pulse and 14.8% of patients showed an absence of radial artery flow when accessed via Duplex ultrasound.^13^ Therefore it’s better to access RAO more objectively using radial flow by performing ultrasound.^14^ RAO may improve with time therefore the incidence of RAO depends on the duration when the radial artery was accessed post-operatively.

Radial artery occlusion is usually higher immediately after the procedure and it reduces with the passage of time. In the PROPHET study, the presence of RAO was 12% after 24 hours which was reduced to half almost when accessed after 28 days (7%).^15^ Radial artery can spontaneously recanalize causing the decline in RAO after some time.

In a prospective study, 2004 patients were checked for RAO after transradial angioplasty after one day at the time of discharge and a 1-month follow-up. At discharge, 93 patients (4.6%) had RAO. But after one-month persistent radial reperfusion was 13% with no oral anticoagulation.^14^ Aykan et al. found a 5.5% frequency of RAO after PCI in their study patients.^10^ Our study showed nearly similar results of 4% RAO 24 hours after the procedure. But there are studies which showed significantly fewer cases of RAO like Voon et al reported no patients with RAO in their study group and there are studies which reported 0.8% RAO.^16^,^17^

However, there are studies which have reported up to 32.9% of cases of RAO after Doppler examination for PCI.^18^ The observations of our studies revealed that RAO has no significant relation to the age gender and BMI of the patient. Munir et al.^19^ found a 11.3% frequency of RAO after PCI in their study patients after trans-radial access. Most of the patients with RAO had a cardiac instability during PCI.

### Limitations:

It includes a simple observational study with single-time Doppler ultrasonography. For a better accessment of RAO after transradial access an analysis with a larger sample size and proper follow-up of RAO via Doppler scan up to 28 days.

## CONCLUSION

The Trans radial approach is associated with fewer complications. RAO is one of the common post-procedural complications. A 24-hour postprocedural accessment is necessary to evaluate the patient for RAO. Objective accessment by colour doppler is of great help in making the right diagnosis.

### Author’s Contribution:

**SNB, MU:** Conceived, designed and did statistical analysis & editing of manuscript.

**IJ, MU:** Did data collection and manuscript writing.

**SNB:** Did review and final approval of manuscript. He is also responsible for the integrity and accuracy of the study.
